# Supplementary material for: Dietary Dityrosine Impairs Glucose Homeostasis by Disrupting Thyroid Hormone Signaling in Pancreatic β-Cells
Source: Foods. 2025 Sep 17;14(18):3220. doi: 10.3390/foods14183220 (PMC12469916; doi:10.3390/foods14183220)
Supplement: Supplementary file 1 [file foods-14-03220-s001.zip › foods-3839669-supplementary.pdf]

## Supplementary Materials

# Dietary Dityrosine Impairs Glucose Homeostasis by Disrupting Thyroid Hormone Signaling in Pancreatic β-Cells

Yueting Ge[1,2,3], Boyang Kou[1], Chunyu Zhang[1], Chengjia Gu[1], Lin Cheng[1,3,4], Yonghui Shi[2], Guowei Le[2*] and Wei Xu[1*]

[1] Henan Key Laboratory of Tea Plant Biology, College of Tea and Food Science, Xinyang Normal University, Xinyang, Henan 464000, China

[2] State Key Laboratory of Food Science and Technology, School of Food Science and Technology, Jiangnan University, Wuxi, Jiangsu Province 214122, China

[3] Dabie Mountain Laboratory, Xinyang, Henan 464000, China

[4] Henan International Joint Laboratory of Tea-oil tree Biology and High-Value Utilization，College of Tea and Food Science, Xinyang Normal University, Xinyang, Henan 464000, China

* Correspondence: Guowei Le, E-mail: lgw@jiangnan.edu.cn

Wei Xu, Email: xuwei@xynu.edu.cn

State Key Laboratory of Food Science and Technology, Jiangnan University, 1800, Lihu Road, Wuxi 214122, China.

Tel.: +86 510 85917789 Fax: +86 510 85869236

# 1. Additional Materials and Methods

*1.1 Chemical Analysis*

Moisture, crude protein and crude fat contents of the freeze-dried pork were determined (in triplicate) by Association of Official Analytical Chemists (AOAC) 950.46, AOAC 990.03 and AOAC 960.39, respectively, in order to adjust and balance the diet ingredients in the mice diets. The results are listed in Supplementary Table S1.

Table S1: Compositional analysis of freeze-dried pork and soy protein isolate.

| Sample | Moisture (%) | Crude protein (%) | Crude fat (%) | Ditry (mg/g) |
|---|---|---|---|---|
| Soy protein isolate | 5.75 ± 0.13 | 91.07 ± 3.25 | - | - |
| LOP | 8.74 ± 0.23 | 66.16 ± 1.80 | 21.37 ± 0.39 | 0.42 ± 0.05 |
| HOP | 5.87 ± 0.16 | 70.04 ± 0.97 | 21.20 ± 0.54 | 0.80 ± 0.09 |

LOP: low-oxidative pork; HOP: high-oxidative pork; Ditry: dityrosine. Data are expressed as mean ± SEM (n=3).

Table S2: Ingredient composition of experimental diets (g/Kg).

| Ingredient | Groups | | | | |
|---|---|---|---|---|---|
| | CON | LOP | HOP | LOP+Dityr | Dityr |
| Soy protein isolate | 211 | - | - | - | 211 |
| LOP | - | 290 | - | 290 | - |
| HOP | - | - | 274 | - | - |
| Dityr | - | - | - | 0.097 | 0.22 |
| Cornstarch | 351 | 351 | 351 | 351 | 351 |
| Maltodextrin | 100 | 100 | 100 | 100 | 100 |
| Sucrose | 100 | 100 | 100 | 100 | 100 |
| Soybean oil | 70 | 70 | 70 | 70 | 70 |
| Pork lard | 90 | 28 | 32 | 28 | 90 |
| Cellulose | 50 | 50 | 50 | 50 | 50 |
| AIN-93G-MX | 35 | 35 | 35 | 35 | 35 |
| AIN-93-VX | 10 | 10 | 10 | 10 | 10 |
| Choline chloride | 2.5 | 2.5 | 2.5 | 2.5 | 2.5 |
| Nutritional level, U/kg | | | | | |
| Energy, kcal | 4529 | 4529 | 4529 | 4529 | 4529 |
| Crude protein[a], g | 192 | 192 | 192 | 192 | 192 |
| Crude fat[b], g | 160 | 160 | 160 | 160 | 160 |
| Carbohydrate, g | 601 | 601 | 601 | 601 | 601 |

[a]Crude protein: diet protein was provided by soy protein isolate in the CON group and freeze-dried pork in the LOP and HOP groups, whose contents were adjusted and balanced according to the measured protein contents in soy protein isolate and freeze-dried pork.

[b]Crude fat: fat contents of each group were adjusted and balanced by adding lard based on the fat content of the soy protein isolate and freeze-dried pork added in the diets.

LOP: Low-oxidative pork, HOP: high-oxidative pork, Dityr: dityrosine. The LOP and HOP diets differed only in the pork cooking methods. The content of Ditry is equal in HOP, LOP + Dityr and Dityr diets. All diets were mainly based on the AIN-93G formulation.

Table S3: Primer sequences for quantitative real-time PCR.

| Genes | Forward (5′–3′) | Reverse (5′–3′) |
|---|---|---|
| β-actin | GGCTGTATTCCCTCCATCG | CCAGTTGGTAACAATGCCATG |
| Nrf2 | AGCACATCCAGACAGACACCAGT | TTCAGCGTGGCTGGGGATAT |
| HO-1 | GAATTCAGCTTGCCACAGGAATTG | TCTACACTAGCTGCATGTTGA |
| NQO1 | AGGATGGGAGGTACTCGAATC | AGGCGTCCTTCCTTATATGCTA |
| TLR4 | TGGATACGTTTCCTTATAAG | GAAATGGAGGCACCCCTTC |
| MyD88 | TCATGTTCTCCATACCCTTGGT | AAACTGCGAGTGGGGTCAG |
| NF-κB | AGCTTATGCCGAACTTCTCG | TGACCCCTGCGTTGGATT |
| TNF-α | CTGAACTTCGGGGTGATCGGT | TCCTCCACTTGGTGGTTTGCTAC |
| IL-6 | ACACACTGGTTCTGAGGGAC | TACCACAAGGTTGGCAGGTG |
| IL-1β | GAAATGCCACCTTTTGACAGTG | TGGATGCTCTCATCAGGACAG |
| IL-10 | CATTCATGGCCTTGTAGACACC | CTTAATGCAGGACTTAAGGGTTA |
| TRβ1 | CCAGAGGTACACGAAGTGTGC | AGGTTTCCAGGGTAACTACAGG |
| MCT-8 | CGGCTGGATAGTGGTGTTTG | CAGAGTTATGGATGCCGAAGATG |
| MafA | CTGGAGGATCTGTACTGGATGA | CGCACGGACATGGATACCA |
| PDX-1 | CTCACCTCCACCACCACCTTCC | CACCTCCTGCCCACTGGCCTTT |
| GLUT2 | ACCCCACTTACAGTCACACCA | CACAGACAGAGACCAGAGCATAG |
| GCK | AGGAGGCCAGTGTAAAGATGT | CTCCCAGGTCTAAGGAGAGAAA |
| Bcl-2 | TCTTTGAGTTCGGTGGGGTCAT | AGACAGCCAGGAGAAATCAAACAGA |
| Bax | CCAGGATGCGTCCACCAAGA | GCAAAGTAGAAGAGGGCAACCAC |
| Caspase-3 | CTGGAGAAATTCAAAGGACGGG | TGAGCATGGACACAATACACGG |

Nrf2, nuclear factor erythroid 2-related factor 2; NQO1, NADPH quinone oxidoreductase 1; HO-1, heme oxygenase-1; TLR4, toll-like receptor 4; MyD88, myeloid differentiation factor 88; NF-κB, nuclear factor kappa beta; TNF-α, tumor necrosis factor-α; IL-6, interleukin-6; IL-1β, interleukin-1β; IL-10, interleukin-10; TR*β*1, thyroid hormone receptor *β*1; MCT-8, monocarboxylate transporter-8; MafA, v-maf musculoaponeurotic fibrosarcoma oncogene homologue A; PDX-1, pancreatic duodenal homeobox-1; GLUT2, Glucose transporter 2; GCK, glucokinase; Bcl-2, B-cell lymphoma 2; Bax, Bcl-2 associated X protein; Caspase-3, cysteinyl aspartate specific proteinase-3.

Table S4: Summary of the major effects of different dietary interventions on metabolic parameters, oxidative stress, inflammation, and thyroid hormone signaling in mice.

| **Parameter / Marker Category** | **Measurement** | **CON group** | **LOP group** | **HOP group** | **LOP+Dityr group** | **Dityr group** |
|---|---|---|---|---|---|---|
| **Metabolic Parameters** | Body weight gain | Baseline | ↔ | ↑↑ | ↑↑ | ↑ |
| | Fasting blood glucose | Baseline | ↔ | ↑↑ | ↑↑ | ↑↑ |
| | Fasting plasma insulin | Baseline | ↔ | ↓↓ | ↓↓ | ↓↓ |
| | Glucose tolerance (AUC) | Baseline | ↔ | ↑↑ | ↑↑ | ↑↑ |
| **Oxidative Damage** | Plasma Dityr, AOPPs, MDA | Baseline | ↔ (MDA ↑) | ↑↑ | ↑↑ | ↑↑ |
| | Pancreatic Dityr, AOPPs, AGEs | Baseline | ↔ (AGEs ↑) | ↑↑ | ↑↑ | ↑↑ |
| **Antioxidant Defenses** | Plasma T-AOC, GSH-Px, SOD | Baseline | ↔ | ↓↓ | ↓↓ | ↓↓ |
| | Pancreatic T-AOC, SOD | Baseline | ↔ | ↓↓ | ↓↓ | ↓↓ |
| | Nrf2/ARE pathway genes | Baseline | ↔ (HO-1 ↓) | ↓↓ | ↓↓ | ↓↓ |
| **Inflammation** | Plasma LPS, TNF-α | Baseline | ↔ | ↑↑ | ↑↑ | ↑↑ |
| | Plasma IL-10 | Baseline | ↓ | ↓↓ | ↓↓ | ↓↓ |
| | Pancreatic TNF-α, IL-1β, IL-6 | Baseline | ↔ (IL-6 ↑) | ↑↑ | ↑↑ (TNF-α ↔) | ↑↑ |
| | Pancreatic IL-10 | Baseline | ↔ | ↓↓ | ↓↓ | ↓↓ |
| | TLR4/NF-κB pathway genes | Baseline | ↔ (NF-κB, IL-6 ↑) | ↑↑ | ↑↑ | ↑↑ |
| **TH Signaling & β-cell Function** | Pancreatic TRβ1 (mRNA/Protein) | Baseline | ↔ | ↓↓ | ↓↓ | ↓↓ |
| | MCT-8 mRNA | Baseline | ↓ | ↓↓ | ↓↓ | ↓↓ |
| | MafA, PDX-1, GLUT2 mRNA | Baseline | ↔ (PDX-1 ↓) | ↓↓ | ↓↓ | ↓↓ (GCK ↓) |
| | Bcl-2 (mRNA/Protein) | Baseline | ↔ | ↓↓ | ↓↓ | ↓↓ |
| | Bax, Caspase-3 (mRNA/Protein) | Baseline | ↔ | ↑↑ | ↑↑ | ↑↑ |
| | Islet area (β-cell mass) | Baseline | ↔ | ↓↓ | ↓↓ | ↓↓ |

↑: Increase compared to CON group; ↓: Decrease compared to CON group; ↔: No significant change compared to CON group. The number of arrows (e.g., ↑↑, ↓↓) indicates the relative magnitude and consistency of the effect across multiple measurements.

LOP: low-oxidative pork; HOP: high-oxidative pork; Dityr: dityrosine; CON: control; AUC: area under the curve; AOPPs: advanced oxidation protein products; MDA: malondialdehyde; AGEs: advanced glycation end products; T-AOC: total antioxidant capacity; SOD: superoxide dismutase; GSH-Px: glutathione peroxidase; LPS: lipopolysaccharide; TNF-α: tumor necrosis factor-alpha; IL: interleukin; TLR4: Toll-like receptor 4; NF-κB: nuclear factor kappa B; TRβ1: thyroid hormone receptor β1; MCT-8: monocarboxylate transporter 8; MafA: v-maf musculoaponeurotic fibrosarcoma oncogene homologue A; PDX-1: pancreatic duodenal homeobox-1; GLUT2: glucose transporter 2; GCK: glucokinase; Bcl-2: B-cell lymphoma 2; Bax: Bcl-2-associated X protein; Caspase-3: cysteinyl aspartate-specific proteinase-3.
